# Social network analysis in football: a systematic review of performance and tactical applications

**DOI:** 10.3389/fpsyg.2025.1659603

**Published:** 2025-09-17

**Authors:** Ricardo Alves, Gonçalo Dias, Nuno André Nunes, Sérgio M. Querido, Vasco Vaz

**Affiliations:** ^1^Faculty of Sport Sciences and Physical Education, University of Coimbra, Coimbra, Portugal; ^2^Polytechnic Institute of Coimbra, Coimbra Education School, Coimbra, Portugal; ^3^SPRINT Sport Physical Activity and Health Research & Innovation Center, Coimbra, Portugal; ^4^Department of Sport and Health, Southampton Solent University, Southampton, United Kingdom; ^5^CIPER, Faculty of Human Kinetics, University of Lisbon, Lisboa, Portugal

**Keywords:** football analytics, social network analysis, tactical behavior, passing networks, performance analysis, team sports, network metrics

## Abstract

**Introduction:**

This systematic review aims to critically examine the application of social network analysis (SNA) in football, with a focus on its contribution to evaluating team performance, tactical behavior, and player interactions.

**Methods:**

Following PRISMA guidelines, a comprehensive search was conducted across four databases (PubMed, Scopus, Web of Science, and SPORTDiscus) from January 2017 to October 2024.

**Results:**

Fifty-five peer-reviewed studies met the inclusion criteria, addressing network analysis in official men's professional football matches. Data were extracted and summarized regarding methodological quality, network metrics used, tactical context, and practical implications.

**Discussion:**

Most studies demonstrated that cohesive network structures, characterized by high density, clustering coefficients, and centrality, are associated with successful team performance. Centrality metrics were frequently used to identify key tactical players, typically central defenders and midfielders. Recent methodological advances included dynamic time-window analysis, pitch-passing networks, and spatial-temporal integration using tracking data. However, there remains an overrepresentation of elite men's football and offensive phases, with limited focus on defensive networks, youth categories, and women's football. SNA offers a powerful framework to decode the complexity of football performance, evolving from static graphs to dynamic, rolesensitive, and context-rich models. Future research should adopt longitudinal designs, multi-layer network approaches, and closer collaboration with practitioners to enhance the operational utility of network insights in coaching and performance analysis.

**Systematic review registration:**

https://osf.io/2pe3y

## 1 Introduction

In professional football, advancements in technology and the continuous evolution of tactical knowledge by coaches, analysts, and researchers have significantly increased the variety and sophistication of methods and metrics used to evaluate team performance (Sarmento et al., 2022). These developments have facilitated the integration of numerous performance indicators and holistic scientific methodologies, essential for objectively analyzing both individual and collective behaviors in modern football (Ribeiro et al., 2017; Sarmento et al., 2022; Mehta et al., 2024). Among these methods, Social Network Analysis (SNA) has emerged as a crucial and complementary framework, offering unique insights into the dynamic patterns of interpersonal coordination within and between football teams (Ribeiro et al., 2017; Alves et al., 2022).

Originally rooted in sociology and anthropology, SNA is an interdisciplinary approach that emerged in the 1930s (Wäsche et al., 2017), grounded in Graph Theory, a mathematical discipline used to examine relationships within groups of actors or organizations (“nodes”) interconnected through ties or links (Rice and Yoshioka-Maxwell, 2015). SNA has since been successfully applied across diverse fields including business, epidemiology, and organizational studies to understand complex interaction systems (Wäsche et al., 2017). The application of SNA in sports science has proven to be successful with several research been identified in the studies of Wäsche et al. (2017). For instance, in basketball it was identified that unpredictability and connectedness across players was associated with better performance (Fewell et al., 2012). In water-polo, through team analysis it was possible to understand attacking strategies and performance outcomes (Passos et al., 2011). Even in motorsport drivers and professional golfers was possible to analyze the relationship between status and its influence on performance (Bothner et al., 2012). In football, nodes represent players, while links depict their interactions, primarily through ball passes (Ramos et al., 2017). These interactions can be visually and mathematically represented via matrices or graphs, revealing the strength and frequency of connections and providing valuable insights into team dynamics and match outcomes (Novillo et al., 2024). Through the application of SNA, practitioners can better interpret their teams “tactical patterns and understand individual players” contributions during matches (Ribeiro et al., 2017).

SNA analyses typically focus on three distinct levels: micro (individual player metrics), meso (small groups of players), and macro (the entire team's network) (Buldú et al., 2018). At the micro-level, key centrality measures include degree centrality, represents the number of passes made by a player (Ramos et al., 2017; Buldú et al., 2018); eigenvector centrality, reflects a player's status within the network (Ramos et al., 2017; Ribeiro et al., 2017); closeness centrality, indicates how quickly the ball circulates among players (Ramos et al., 2017; Ribeiro et al., 2017; Buldú et al., 2018); and clustering coefficient, measures how well-connected a player is with their immediate neighbors, often reflected by the ability of players in creating triangles (Peña and Touchette, 2012; Ramos et al., 2017; Ribeiro et al., 2017; Buldú et al., 2018). At the meso-level, prominent metrics include average neighbor degree, which assess interaction strength between player pairs; assortativity coefficient, identifies the frequency of interactions among highly influential players; and topological overlap, captures player groupings based on shared connections (Clemente et al., 2016). Lastly, macro-level analysis provides an overarching perspective of the team's collective interactions, featuring metrics such as total links (overall interactions), network density (overall team cohesion), distance (speed of ball circulation), network diameter (maximum interaction distance), clique (frequency of specific interaction patterns), network heterogeneity (variety of connections), centralization (distribution of team interactions), and global prestige (overall prominence of the team) (Oliveira and Gama, 2012; Clemente et al., 2016; Ribeiro et al., 2017; Ievoli et al., 2021b).

Research has demonstrated practical implications of these metrics; for instance, high clustering coefficients can indicate effective utilization of central areas, notably by central midfielders, as exemplified by Spain's successful tactics in the 2010 FIFA World Cup (Cotta et al., 2013). Metrics like network density are predictive of goal-scoring potential and overall team success, aiding coaches and analysts in identifying effective game models and influential players (Grund, 2012; Pina et al., 2017). Furthermore, SNA applied to training exercises has revealed that teams composed of more skilled players exhibit higher levels of network cohesion and connectivity, enabling tailored adjustments to training methodologies (Machado et al., 2021; McHale and Relton, 2018).

Most of the analytical methods described above are commonly referred to as *player-passing network*, with focus mainly at individual analysis. Recent methodological developments have introduced alternative approaches, such as *pitch-passing networks* and *pitch-player passing networks* (Buldú et al., 2018). The *pitch-passing network* involves in the dividing into predefined zones, where nodes represent these zones and links the passes between them (Buldú et al., 2018). The *pitch-player passing network*, it's a combination of a player and its position in the moment of the pass (Buldú et al., 2018). This integrative method has proven particularly insightful in case studies, e.g., FC Barcelona, which exhibited distinct patterns compared to other teams in La Liga. The team showed a higher number of triangular connections between pitch zones, indicating greater robustness, and elevated clustering coefficient values, reflecting fluid ball circulation across multiple zones (Herrera-Diestra et al., 2020). Although this method offers a more holistic perspective of team behavior, its application remains dependent on the complexity of data collection and computational analysis.

Like any other approach, SNA metrics requires careful contextualization with established situational variables known to influence football performance such as, match status, quality of opponent, time windows, match location and tactical systems (Mclean et al., 2018a; Praça et al., 2019; Aquino et al., 2020; Pan et al., 2024; Mclean et al., 2019). For example, teams may adopt a more direct style of play in losing situations, in contrast to winning scenarios where possession-based approach is more common (Praça et al., 2019). Furthermore, winning contexts tend to increase the prominence of midfielders, wingers and forwards within the network structure, reflecting with greater involvement in maintaining possession and creating offensive opportunities (Praça et al., 2019). Moreover, tactical systems also play a crucial interpretative role in the analysis of network metrics. Aquino et al. (2019) reported that the 1-4-2-3-1 tactical system was associated with higher macro- and micro-level network metrics when compared to the 1-4-4-2 and 1-4-3-3 tactical systems, suggesting greater cohesion and structural connectivity. Such findings can be further interpreted in the light of individual positional roles, for instance, forwards may exhibit higher passing volumes in a 1-4-2-2-2 tactical system, while defensive midfielders tend to present elevated betweenness centrality values in a 1-4-2-3-1 tactical system, reflecting their function as possession anchors (McLean et al., 2018b). From a practical perspective these patterns are also shaped by playing style and player-specific attributes (Diquigiovanni and Scarpa, 2019). For instance, Díaz Díaz et al. (2024) observed that central defenders and midfielders in the Argentinian national team displayed high connectivity metrics such as Degree Centrality and Closeness Centrality, while highly influential players like Messi were the most frequently recruited, presenting higher values in Page Rank, Eigenvector Centrality, Hubs and Authority. Together, these insights underscore that tactical context and player profiles interact to shape network structures, highlighting the need to account for both systemic and individual factors when interpreting SNA metrics in football. With the ongoing tactical evolution of football, characterized by increasingly hybrid systems, players are now required to demonstrate high adaptability to perform effectively across varying positional and structural demands.

SNA has become central to football research, particularly regarding collective tactical actions. Its growing adoption underscores not only theoretical significance but also substantial practical utility, informing tactical decisions, training practices, and overall performance optimization. Previous reviews have addressed network analysis applications in football and sports broadly (Sarmento et al., 2014, 2018; Wäsche et al., 2017; Caicedo-Parada et al., 2020), yet the rapid expansion of research post-2019 necessitates an updated and more comprehensive synthesis. New research since 2020 has significantly expanded analytical methods, covering broader competitive contexts, various age groups, genders, tactical formations, and game phases, further justifying the timeliness and necessity of this systematic review.

Recent methodological advances have introduced additional network metrics such as, proximity prestige, betweenness centrality, entropy, variability, and robustness (Herrera-Diestra et al., 2020; Martínez et al., 2020; Martins et al., 2020, 2021; Sarmento et al., 2020; Ichinose et al., 2021; Alves et al., 2022; Gama et al., 2020; Gonçalves et al., 2021; Medina et al., 2021). Furthermore, contemporary studies increasingly employ machine learning, multilayer networks, and real-time data collection technologies (e.g., GPS and multi-camera systems) (Korte et al., 2019; Ievoli et al., 2021a; Pan et al., 2024; Ievoli et al., 2021b; Zhang et al., 2018). This surge in methodological sophistication reflects a concerted effort to bridge scientific analysis with practical coaching applications, enhancing practitioners' understanding of player influence, collective efficacy, and tactical decision-making.

Consequently, this systematic review aims to comprehensively examine recent literature on network analysis in football, highlighting critical themes, methodological innovations, and practical implications. By doing so, it seeks to contribute significantly to the theoretical and methodological advancement in football analytics, fostering a critical understanding of current research trends and guiding future investigations and practical applications.

## 2 Materials and methods

### 2.1 Design

This systematic review on network analysis in football adhered strictly to the “Preferred Reporting Items for Systematic Reviews and Meta-Analyses” (PRISMA) guidelines (Page et al., 2021), following the minimum methodological standards outlined by the Cochrane Back Review Group (Furlan et al., 2009). Prior to conducting the literature search and selection procedures, a detailed review protocol based on PRISMA-P guidelines (Page et al., 2021) was developed (see Supplementary Table—Prisma). The protocol was registered on Open Science Framework (https://osf.io/).

### 2.2 Literature search and selection process

Literature search and selection process a comprehensive and systematic literature search was conducted across four electronic databases: PubMed (MEDLINE), SPORTSDiscus, Scopus, and ISI Web of Science (WOS). The search strategy incorporated Boolean operators (AND, OR, NOT) to combine the primary keywords “network” and “football” along with relevant synonyms. The specific search strategies were adjusted to each database's unique characteristics and are detailed in the Appendix (Search Strategies Adopted).

In addition to the electronic searches, reference lists from included studies were manually reviewed to identify further relevant articles. All searches and subsequent screening processes were independently carried out by two authors (RA and GD) from October 2 to the end of March 2025. Any discrepancies in article selection were resolved by consensus. To minimize the possibility of missing pertinent studies, two co-authors with expertise in network analysis and football directly contributed to the data processing and interpretation phases of the study.

Eligibility criteria for study selection were structured according to the PICOS framework:

1. Population: Football teams;
2. Intervention: Network analysis methods frequently applied in existing studies;
3. Comparison: Comparative analyses between different network analysis methods;
4. Outcome: Results specifically related to network analysis in football;
5. Study design: Studies utilizing network analysis in various competitive contexts and innovative network methods.

Inclusion criteria stipulated that studies must be peer-reviewed, written in English, and include data from official 11-a-side football matches involving adult male football players. Exclusion criteria included studies featuring non-football players or unofficial matches, unrelated sports, youth athletes (<18 years old), female athletes, conference abstracts, or those lacking relevant network analysis data.

Identified titles and abstracts were exported into Mendeley Desktop (version 1.19, Glyph and Cog, London, United Kingdom) and screened against the eligibility criteria (Querido et al., 2022). Following duplicate removal and initial screening, eligible full-text articles were independently reviewed and selected based on predefined criteria (Querido et al., 2022). Data extraction was independently conducted by one researcher, and any doubts about a specific article would be resolved through consensus by two researchers (RA and GD). The extracted data included the general characteristics and primary findings of each study, which were systematically summarized in both narrative form and structured tables (Querido et al., 2022).

## 3 Results

### 3.1 Search results

**Figure 1:**
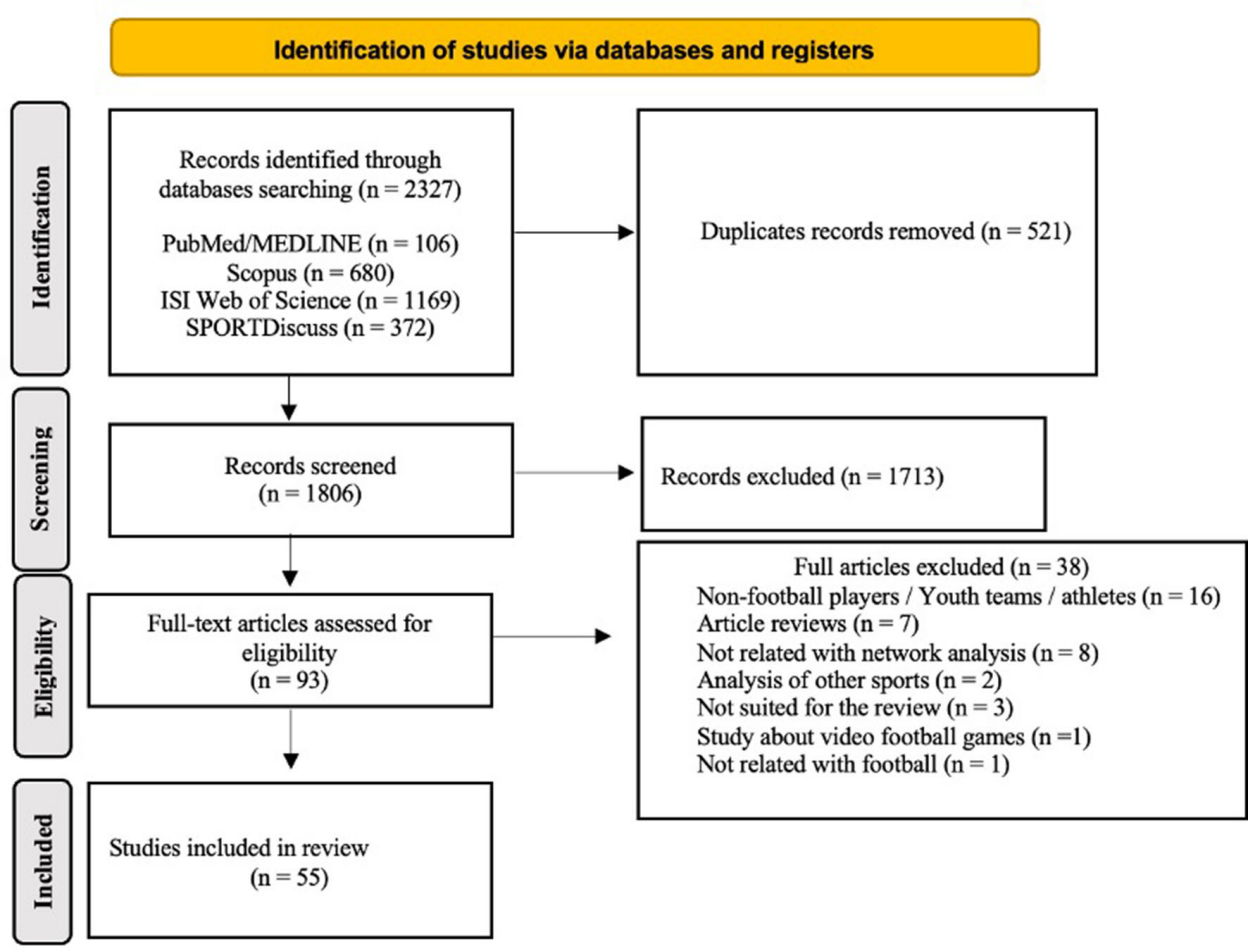
Flowchart illustrating the systematic review process of identification, screening, eligibility, and inclusion of studies.

The systematic literature search was conducted between 1st January 2017 and October 2024. Initially, 2,327 articles were retrieved across the four selected databases (PubMed/MEDLINE, Scopus, ISI Web of Science, SPORTDiscus). After removing duplicates (*n* = 521), 1,806 records underwent title and abstract screening. At this stage, 1,713 articles were excluded due to irrelevance to the research objectives. The remaining ninety three full-text articles were thoroughly assessed for eligibility, resulting in the exclusion of an additional thirty-eight studies based on the following reasons: non-football or youth athletes samples (*n* = 16), review articles (*n* = 7), irrelevant to network analysis (*n* = 8), analyses of other sports (*n* = 2), unsuitable methodologies or data (*n* = 3), studies on football video games/eSports (*n* = 1), and not specifically related to football or soccer (*n* = 1). Ultimately, fifty five studies were included for qualitative synthesis and methodological quality assessment (Figure 1).

### 3.2 Data collection and analysis

A Microsoft Excel spreadsheet (Microsoft^®^, USA) was created to capture all relevant data such as, Authors, Year and Journal of Publication, Sample, Country of Origin, Competition, Networks Metrics Category, Networks Variables used and the general Key Findings.

All data analysis and visualization graphics were made in RStudio (version 2023.06.1).

### 3.3 Methodological quality assessment

The methodological quality and risk of bias of the included studies were evaluated using a structured checklist composed of multiple criteria distributed across clearly defined subscales (detailed criteria provided in Supplementary Table 2). Two reviewers (RA and GD) independently assessed each study's methodological quality according to guidelines outlined by Sarmento et al. (2018). Any discrepancies were resolved through consensus.

The methodological quality of the reviewed studies was categorized as follows:

- Low quality: score ≤ 50%
- Good quality: score between 51% and 75%
- Excellent quality: score above 75%

The average methodological quality across all included studies was 73.9%, with no study scoring below 50%. Specifically, 65% (*n* = 36) of studies were classified as excellent, while 35% (*n* = 19) were classified as good quality. Notably, none of the studies reached the maximum score of 100%.

### 3.4 Summary of included studies

A comprehensive summary detailing each included study's author(s), publication year, journal, sample characteristics, research aims, variables (social network analysis metrics), and key findings is provided chronologically in Supplementary Table 1.

#### 3.4.1 Publication distribution

**Figure 2:**
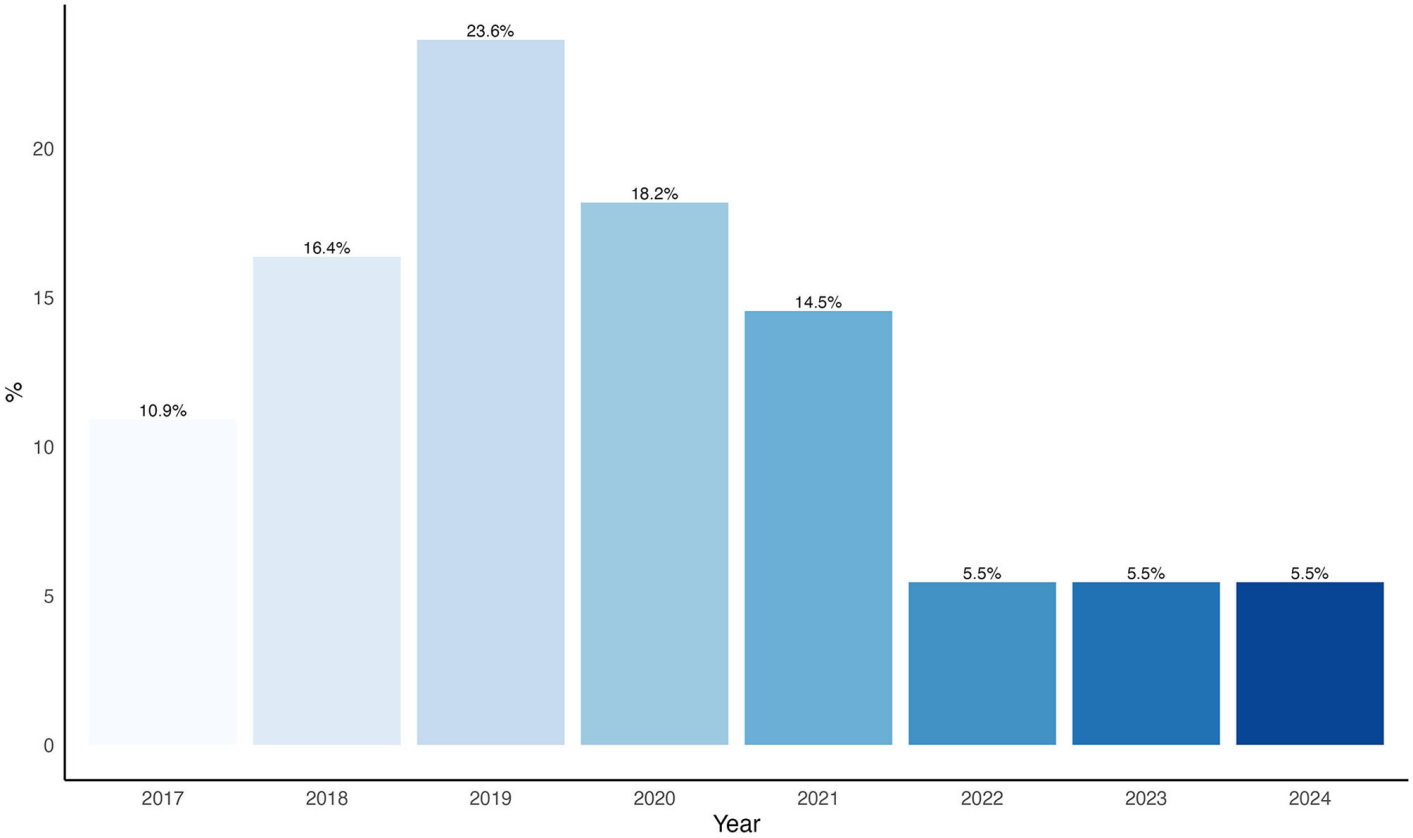
Percentage of studies by year.

The distribution of articles by publication year demonstrated increasing research activity in recent years, showing an interest in this area (Figure 2).

**Figure 3:**
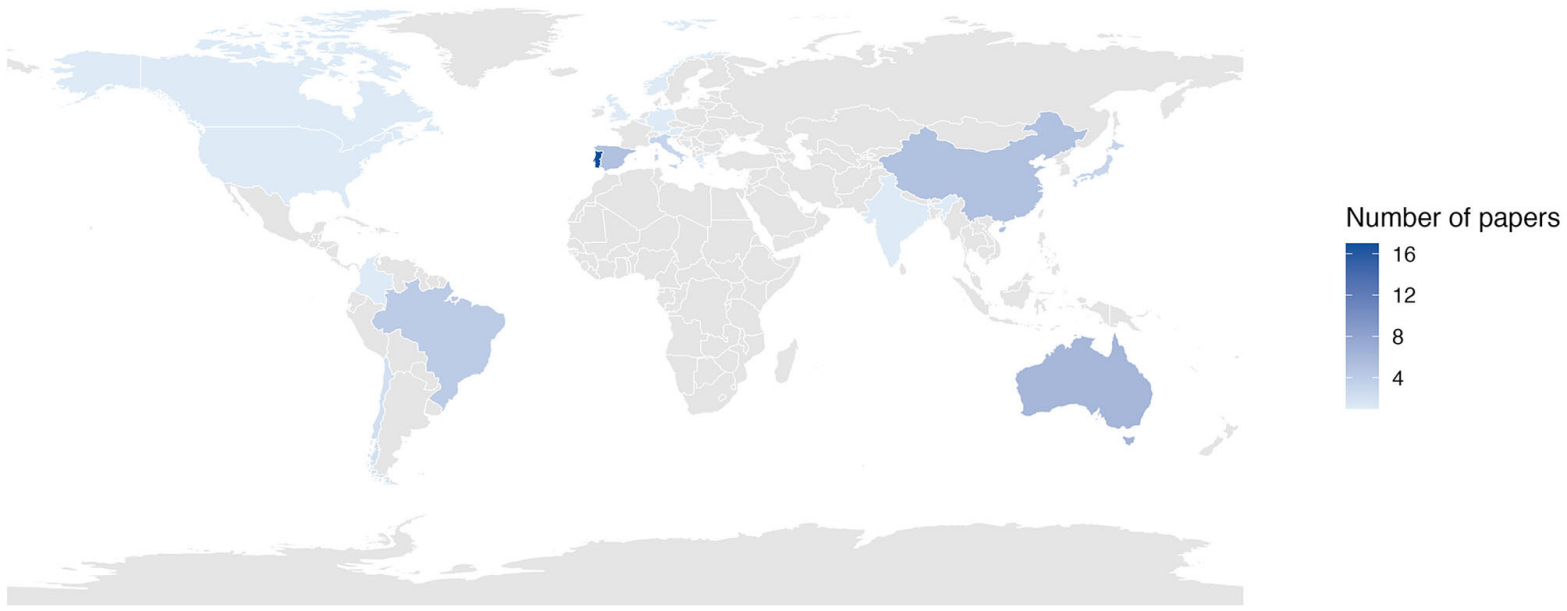
Heatmap illustrating the distribution of publications by country.

Moreover, regarding the country of origin of the publications (Figure 3), Portugal accounted for the largest proportion of studies (30.9%), followed by Australia (10.9%), China (9.1%) and Spain (9.1%).

#### 3.4.2 Journals with highest contributions

**Figure 4:**
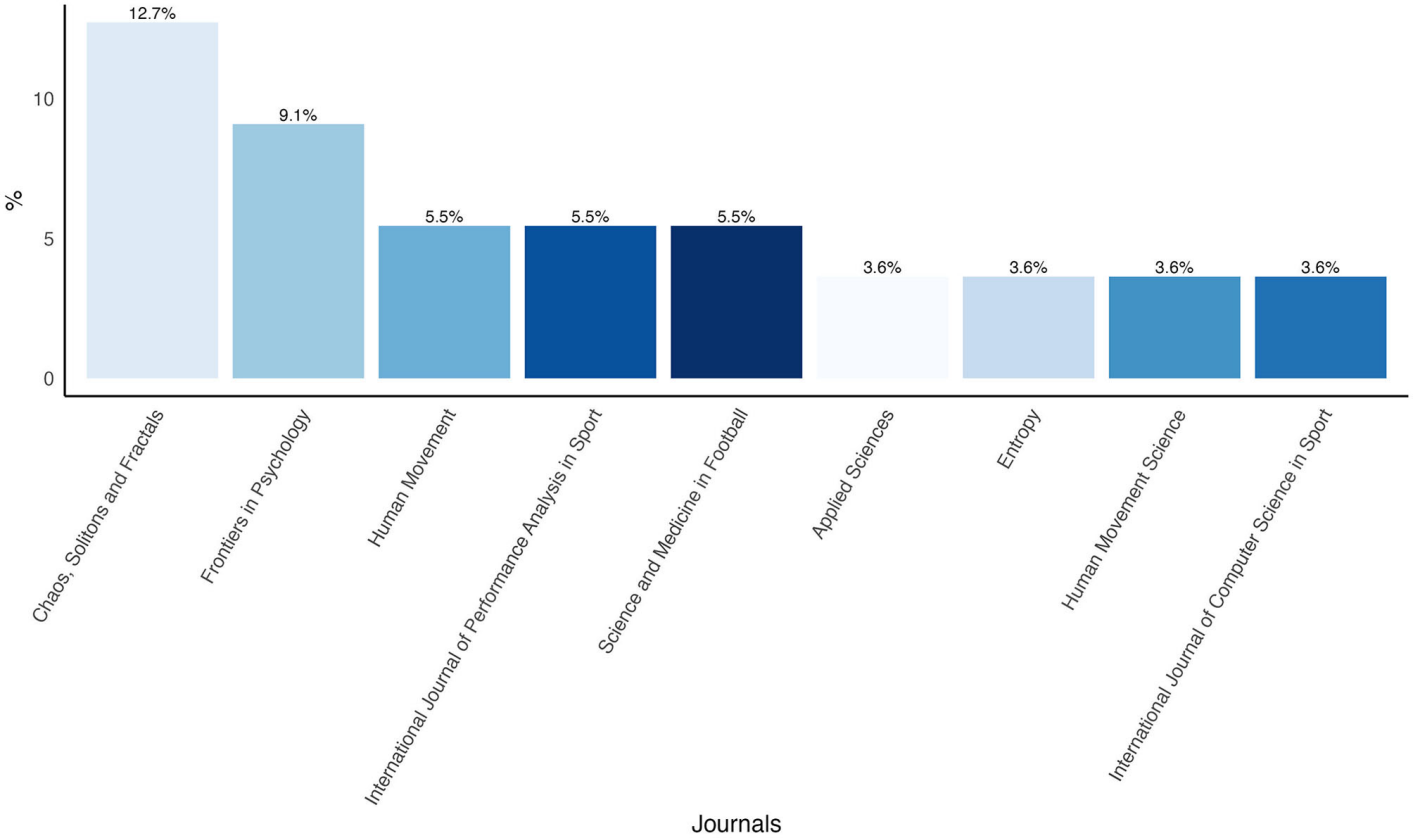
Percentage of the top 9 most publishing journals.

The journals with the highest proportion of reviewed articles included were *Chaos, Solitons and Fractals* (12.7%) and *Frontiers in Psychology* (9.1%). Figure 4 displays the top nine journals in terms of publication frequency.

#### 3.4.3 Competitive context

**Figure 5:**
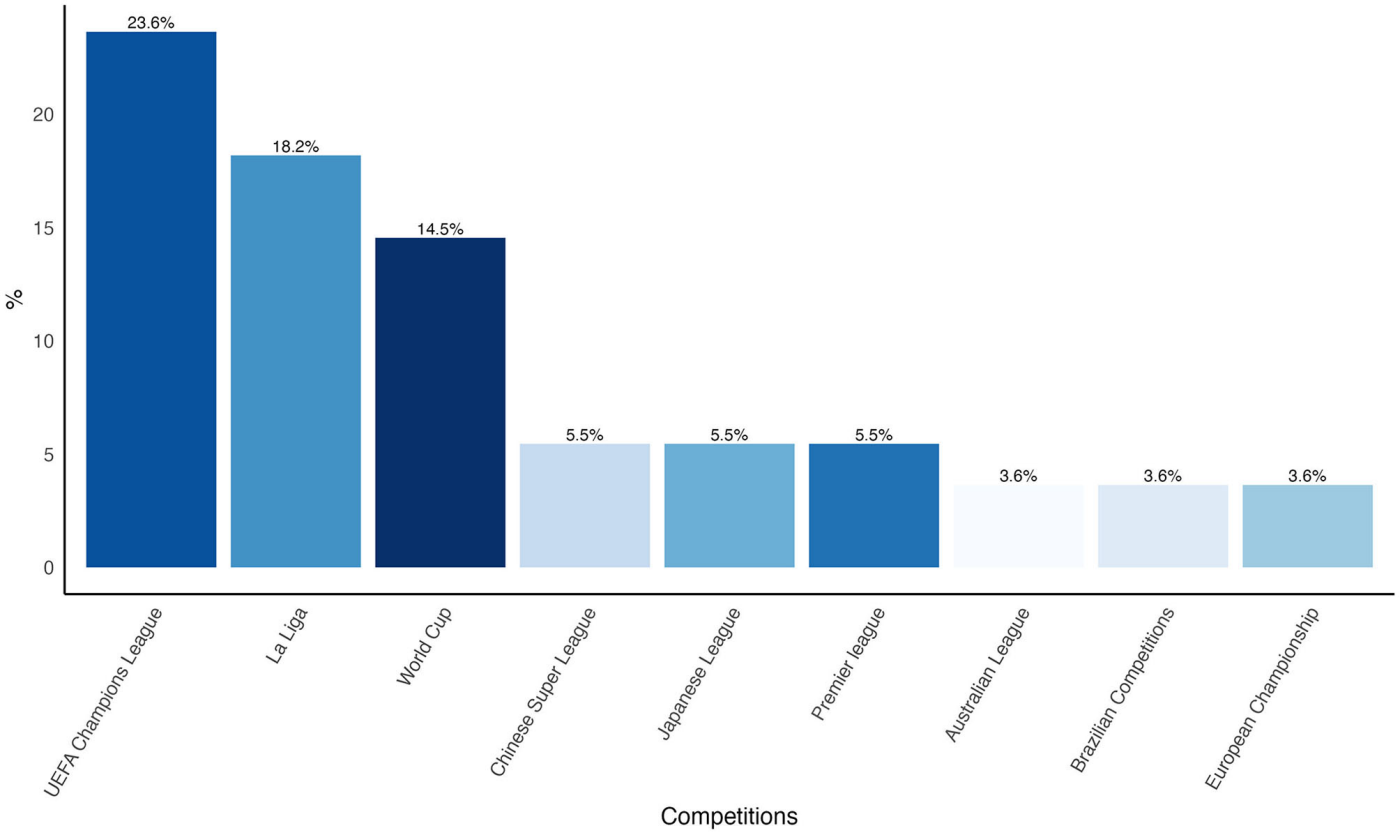
Percentage distribution of the nine most frequently analysed competitions across the reviewed articles.

Studies predominantly analyzed high-level competitions, including UEFA Champions League (23.6%), La Liga (18.2%) and FIFA World Cup (14.5%). Figure 5 displays the nine most analyzed competitions in the reviewed articles.

Typically, studies either examined multiple matches within specific competitions or focused on detailed analyses of successful teams within these competitions. There's a lack of studies who focused in analyzing a specific team in a longitudinal perspective.

#### 3.4.4 Social network analysis metrics category

**Figure 6:**
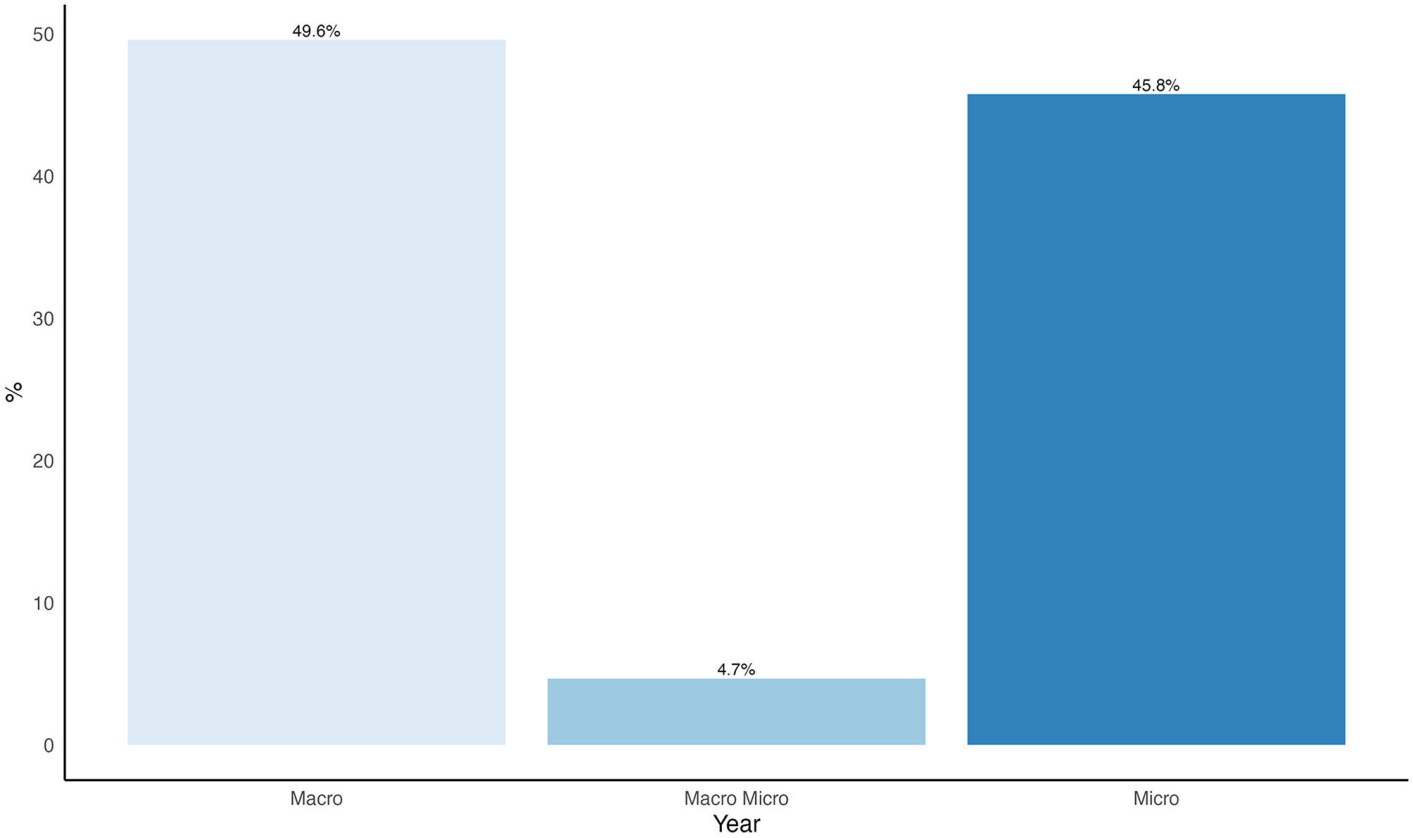
Percentage distribution of the analysed network metrics.

A variety of network analysis metrics were identified, including network density, betweenness centrality, clustering coefficient, among others. Based on these metrics, the studies were categorized (Figure 6) according to the analytical level employed: Macro-level analysis (49.6), Micro-level analysis (45.8%) and Meso-level analysis (4.66%) (integrating both micro and macro analytical perspectives).

#### 3.4.5 General overview of individual studies

The objectives of the reviewed studies were diverse, with a primary focus in analyzing players interactions and team dynamics. Several studies aimed to recognizing performance patterns, including goal scoring action and opportunities, connection between pitch zones (pitch passing network) and the identification of key players within the team structure.

**Figure 7:**
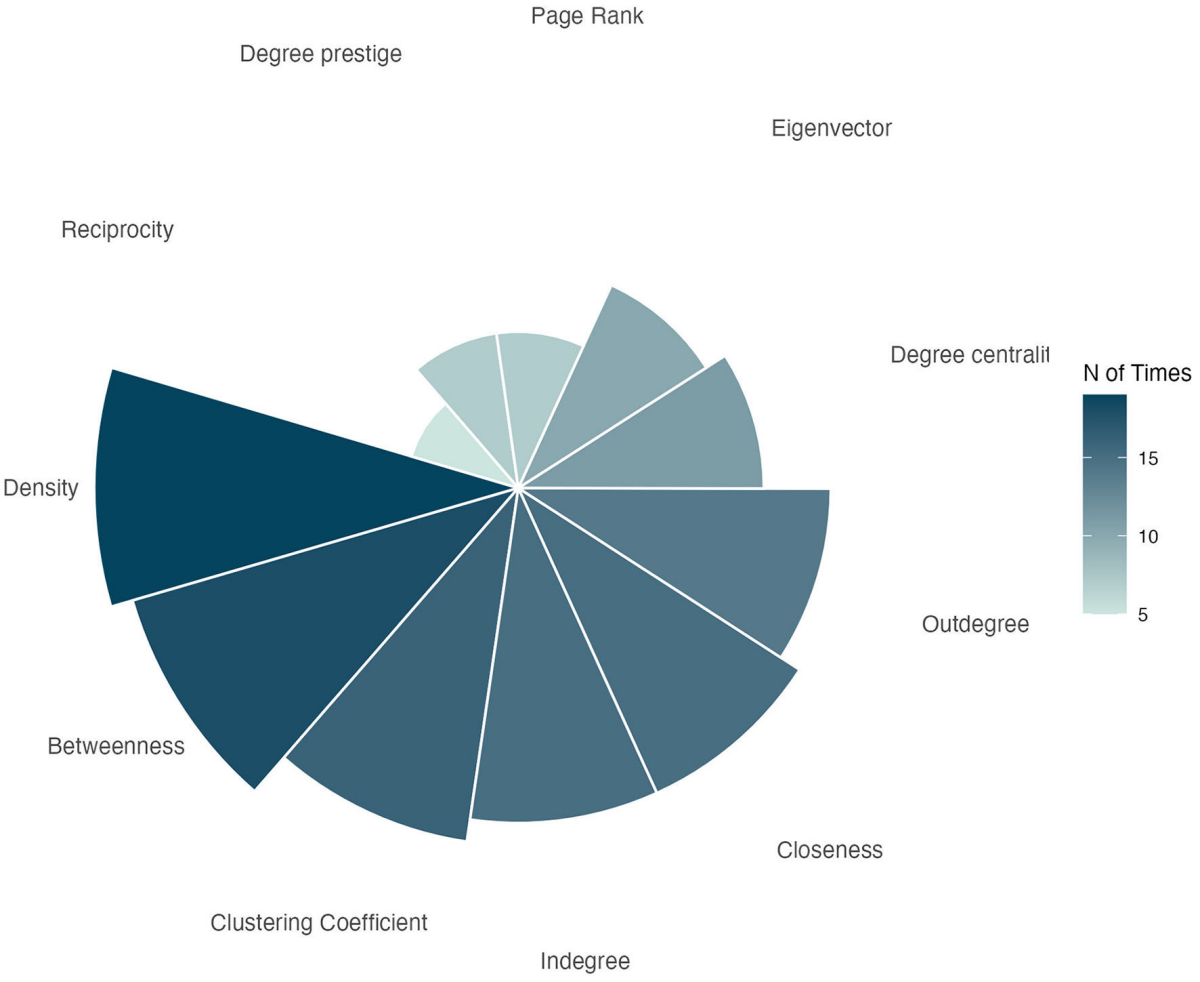
Variables frequency used in the studies.

As previously mentioned, a wide range of network metrics was employed across the studies. Among the most frequently used were network density, betweenness centrality, clustering coefficient, and closeness centrality, which are predominantly associated with macro-level or team-level perspective. In contrast, indegree and outdegree metrics—commonly associated to the passing interactions—were frequently employed to assess the individual roles and contributions of players within the team's passing network (Figure 7).

**Figure 8:**
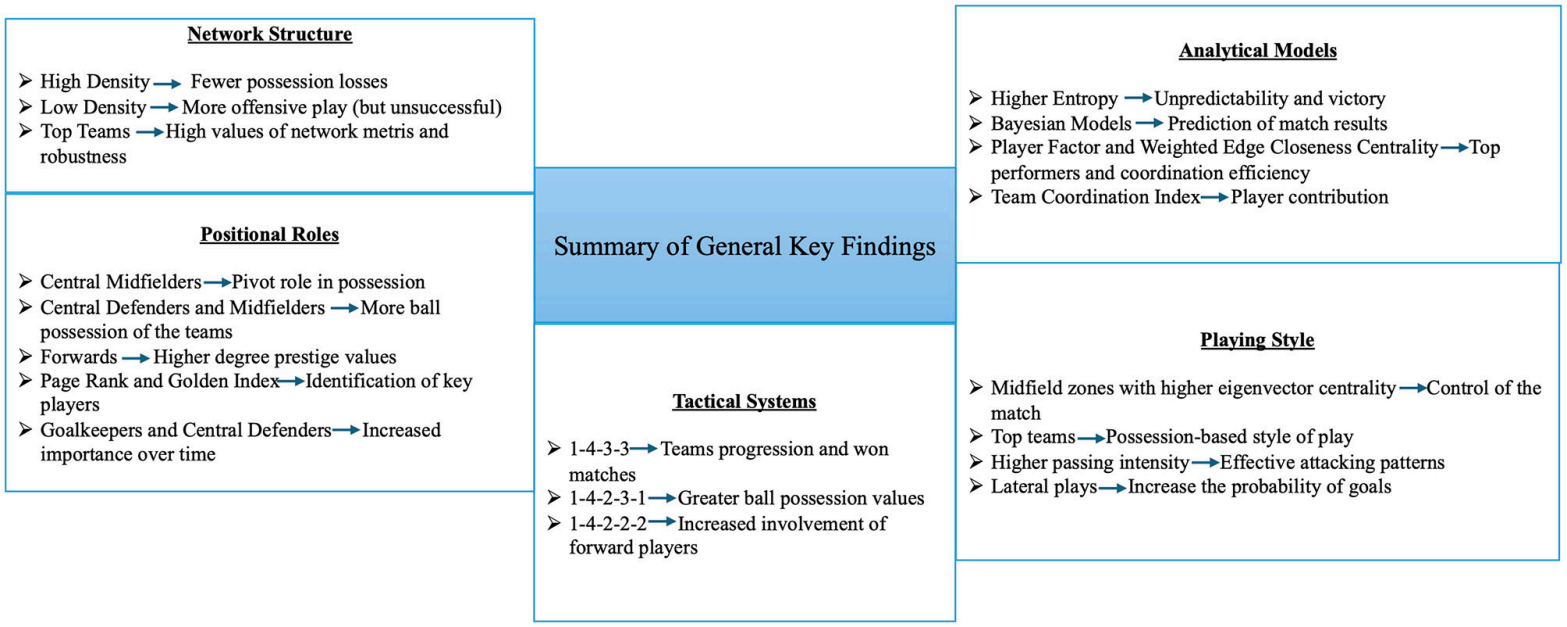
Summary of general key findings.

In the analyzed studies, there are certain key findings that standout and be grouped in the following categories: network structure, positional roles, playing style, tactical systems and analytical models. These categories can group some of the insights of the analyzed studies (Figure 8).

This detailed synthesis offers a structured representation of the current research landscape on social network analysis in professional football, clearly delineating trends in methodological approaches, analytical focus, and quality benchmarks.

## 4 Discussion

In response to the growing need for updated analytical approaches in football, this systematic review aimed to synthesize current research using network analysis as a tool to evaluate team performance and tactical behavior. Drawing from fifty five peer-reviewed articles published between 2017 and 2024, this section discusses prevailing trends, methodological progressions, and practical implications of the use of network analysis in football match analysis.

### 4.1 Performance context and network structure

A consistent finding throughout the literature is the positive association between cohesive network structures and enhanced team performance. Multiple studies highlight that teams with highly interconnected passing networks tend to demonstrate superior ball retention, fluid transitions during possession, and more effective offensive actions (Clemente and Martins, 2017a; Pina et al., 2017; Kawasaki et al., 2019). Specifically, winning teams have shown greater local interconnectivity (as indicated by higher clustering coefficients) suggesting a strategic emphasis on short-passing sequences among proximate players (Aquino et al., 2019; Medina et al., 2021). Similarly, top-ranked teams displayed higher levels of network density, average degree, weighted degree, and clustering coefficients (Pan et al., 2024; Clemente, 2018; Castellano and Echeazarra, 2019; McLaren and Spink, 2020), reinforcing the link between structural connectivity and performance outcomes.

However, the relationship between network metrics and success is not universally straightforward. For instance, Mclean et al. (2018a) indicated no significant differences in passing network characteristics between successful and unsuccessful teams, nor between group and knockout stage performances in the UEFA EURO 2016 tournament. This suggests that performance metrics may be influenced by additional contextual or tactical factors.

Furthermore, several studies reported tactical shifts in response to match status. Praça et al. (2019) found that teams tended to adopt a more direct playing style when leading, while those trailing often favored a possession-based approach (reflected in changes at the microstructural level). These adaptations highlight the dynamic nature of team strategies and their influence on network properties (Vivés et al., 2018; McLean et al., 2017; Yamamoto and Narizuka, 2018; Zhao and Zhang, 2020).

Nonetheless, researchers have cautioned against over interpreting network metrics in isolation. Aquino et al. (2019) emphasized that performance success can emerge from a variety of tactical models, and thus variability in both macro- and micro-level network measures is not uncommon. This underlines the importance of contextualizing network data within broader match dynamics and strategic objectives.

### 4.2 Team and player analysis

Network analysis in football typically operates on two levels: team-level and player-level. At the team level, network metrics provide an overarching view of how players are connected, offering valuable insights into the team's tactical structure and playing style. For instance, longitudinal analyses of World Cup tournaments have demonstrated how variations in style affect network density. In 2018, a more direct style of play was evident, reflected by lower density values, suggesting fewer connections between players. By contrast, the 2022 World Cup showed a shift towards a possession-based style among top-tier teams, characterized by higher density metrics (Pan et al., 2024).

Such findings suggest that stylistic preferences—shaped by competition context, age, or gender—can influence network structure, which in turn should inform training design and match preparation (Armatas et al., 2022; Oliveira and Clemente, 2018). Key metrics such as density and clustering coefficient have been consistently used to assess overall team cohesion and the extent to which players maintain local connections with teammates (Clemente and Martins, 2017a; McLean et al., 2018b; Immler et al., 2021; Mclean and Salmon, 2019). Successful teams tend to exhibit higher engagement in subgroups and greater overall connectivity (Clemente and Martins, 2017a; Immler et al., 2021). In fact, network density has been proposed as a potential predictor of success, as it reflects the structural integration of team interactions (Pina et al., 2017). Guardiola's teams, for instance, have demonstrated higher density values, which have been associated with prolonged ball possession and intricate passing sequences (Immler et al., 2021).

At the player level, centrality metrics are commonly used to identify individuals who serve as tactical hubs. Degree centrality captures the frequency of a player's involvement in passing exchanges and often highlights central defenders and midfielders as dominant contributors (Peixoto et al., 2017). However, when considering degree prestige, which identifies how frequently a player is targeted, results are more nuanced. In some cases, strikers attain high prestige values due to their critical positioning near the goal, despite their limited involvement in build-up play (Clemente and Martins, 2017b; Clemente et al., 2019, 2020; Korte et al., 2019; Sarmento et al., 2020; Yu et al., 2020; McLean et al., 2021; Alves et al., 2022).

Additional metrics such as betweenness centrality, clustering coefficient, closeness centrality, eigenvector centrality, and proximity prestige offer more nuanced insights into player influence and strategic positioning (Arriaza-Ardiles et al., 2018; Buldú et al., 2019; Korte et al., 2019; Wiig et al., 2019; Martins et al., 2021; Assunção et al., 2022; Nath and Chowdhury, 2024). Innovative tools like the Golden Index have also been developed to quantify players' offensive contributions, identifying key individuals driving team performance (Pereira et al., 2019b,a).

These findings underscore the importance of interpreting metrics within their tactical context. Rather than relying solely on statistical outputs, analysts must consider player roles, spatial positioning, and strategic objectives when evaluating network data. A player's prominence in the network is ultimately a function of the team's tactical configuration and the demands of their specific role (Clemente et al., 2019; Alves et al., 2022).

### 4.3 Impact of tactical systems on network analysis

While tactical formations are a central aspect of football strategy, relatively few studies have explicitly examined their influence on network metrics. Nevertheless, emerging evidence suggests that tactical systems can significantly shape both team-level and positional network structures. For example, teams operating in a 1-4-2-3-1 formation have been found to exhibit higher values in both micro and macro-level network metrics when compared to those using 1-4-4-2 or 1-4-3-3 systems, indicating greater cohesion and structural connectivity (Aquino et al., 2019).

Positional contributions also appear sensitive to tactical arrangements. Forwards demonstrated higher passing volumes when their teams played in a 1-4-2-2-2 system compared to a 1-4-2-3-1, suggesting increased involvement in build-up play within that shape. Conversely, defensive midfielders showed a higher proportion of betweenness centrality in the 1-4-2-3-1 formation, indicating their enhanced role as intermediaries in linking play (McLean et al., 2018b).

These findings imply that subtle modifications to tactical systems can influence the prominence and effectiveness of certain positions. Coaches can use this information to align tactical choices with the technical profiles and strengths of their players, thereby enhancing team cohesion and performance.

Future research should aim to integrate more detailed tactical descriptors, such as pressing strategies, width utilization, or defensive compactness, into network analyses. Doing so would strengthen the connection between empirical findings and applied coaching practice, offering deeper, context-sensitive insights for optimizing performance.

### 4.4 New methods to analyze the network

To better understand player positioning and passing structure, recent studies have introduced advanced methodological approaches that enhance the traditional static view of network analysis. One such innovation involves calculating the average origin of passes using x and y coordinates, which allows for a more graphical and spatially contextualized visualization of team networks (Zhou et al., 2023). Furthermore, the integration of composite measures, such as the Pezzali score, enables a more nuanced understanding of coordinated team performance by combining multiple network features (Zhou et al., 2023).

Traditional network analyses often take a static approach, aggregating data across an entire match. However, football is inherently dynamic, and the use of sliding time windows (e.g., 2- or 5-m intervals) allows researchers and practitioners to examine the evolution of passing structures and detect real-time shifts in team strategy (Cao, 2023). Combined with graph distance measures, this approach provides a clearer view of how network structure adapts across different phases of play.

A recognized limitation of classic network models is their lack of spatial specificity. To address this, a growing number of studies have introduced pitch-passing networks, which incorporate spatial dimensions by dividing the field into multiple zones (nodes) and quantifying the volume of passes between them (links) (Buldú et al., 2019; Herrera-Diestra et al., 2020; Gong et al., 2023; Novillo et al., 2024). This method not only enhances the spatial resolution of passing analysis but also enables comparison across teams and leagues. For instance, pitch-passing networks have revealed that teams finishing at the top of league tables tend to adopt more possession-oriented styles compared to lower-ranked teams (Gong et al., 2023).

In comparison to player-based passing networks, pitch-passing networks offer a more system-level perspective on offensive behavior, revealing how collective ball movement unfolds across the field. When combined with time window analysis, this dynamic approach allows coaches and performance analysts to monitor and respond to in-game tactical changes more effectively (Cao, 2023).

Finally, emerging experimental studies are also integrating network metrics into player performance models, providing new tools to assess individual influence and identify tactically critical players within specific formations or match contexts (Nath and Chowdhury, 2024). These developments underscore the evolving potential of network analysis as a diagnostic and strategic resource in elite football.

### 4.5 Limitations

The present systematic review has several limitations that should be acknowledged. First, the included studies primarily focus on professional-level male football, excluding youth categories and women's football. Incorporating a broader range of samples, including different age groups and female athletes, could reveal important variations in network behaviors and offer a more inclusive understanding of tactical patterns across different footballing contexts.

Second, only a small number of studies analyzed data from an entire season for a single team. As previously noted by Caicedo-Parada et al. (2020), analyzing full-season data is essential to identify longitudinal fluctuations in network structures and tactical dynamics. Although recent studies have begun to address this gap (Alves et al., 2025; Da Conceição Alves et al., 2025), the existing literature remains skewed towards cross-sectional or tournament-based analyses. This is compounded by an overrepresentation of certain competitions, particularly European leagues and national team tournaments, limiting the generalizability of findings to other footballing cultures and systems.

Additionally, many studies rely on limited sample sizes, often based on single-match analyses, which restrict the ability to detect broader tactical trends or structural consistencies. Another critical gap in the literature is the predominant focus on offensive network patterns. Defensive behaviors, though equally vital in shaping match outcomes, have received minimal attention. Future research should consider integrating defensive metrics into network frameworks, particularly under match conditions, as emerging studies in small-sided and conditioned games have started to explore (Pacheco et al., 2022).

Addressing these limitations in future research will enhance the robustness and applicability of network analysis in football performance contexts.

### 4.6 Practical application and future research

The practical relevance of network analysis in football has grown considerably in recent years, with increasing emphasis on its operational utility for coaches, analysts, and support staff. Centrality metrics, for example, offer valuable insights into how individual players contribute to collective team performance, aiding in tactical evaluations and player-specific feedback.

Advancements in tracking technologies and the availability of large-scale spatiotemporal datasets have further enhanced the potential of network analysis. These tools now enable real-time assessment of passing networks, allowing practitioners to monitor how structural dynamics evolve throughout a match. The integration of event data (e.g., passes, shots) with positional data (e.g., player coordinates) presents an opportunity to generate more sophisticated and actionable insights, both at the individual and collective levels.

Moreover, this convergence of data streams may facilitate the development of new metrics that better reflect tactical roles, decision-making processes, and situational variables. For example, combining network measures with context-specific indicators (e.g., match status, opposition pressure) could yield deeper understanding of team behavior under different scenarios.

Despite these advancements, a gap remains between scientific research and practical application. As highlighted by Plakias et al. (2025), the translation of network findings into coaching practice is still limited. Key challenges include the development of intuitive visualization tools, accessible and user-friendly software platforms, and the alignment of network metrics with performance indicators familiar to technical staff.

Future research should focus on bridging this gap by co-developing tools and frameworks in collaboration with coaches, ensuring that scientific outputs are directly relevant, interpretable, and usable within real-world football environments. Emphasizing applied relevance will be crucial in maximizing the impact of network analysis on team preparation, tactical planning, and match-day decision making.

## 5 Conclusion

Network analysis has emerged as a powerful framework for understanding the complexity of modern football, offering valuable insights for coaches, performance analysts, and researchers alike. By updating and extending previous reviews, this systematic review critically synthesizes current evidence while providing a robust conceptual and methodological foundation for future developments in the field. Through a comprehensive search of multiple databases, the most relevant studies were identified and analyzed to inform both academic research and applied practice.

Recent advancements in data availability, tracking technologies, and analytical methodologies have significantly expanded the scope of network analysis. Novel metrics such as entropy, pitch-passing networks, the Golden Index, and network robustness enable more dynamic, context-sensitive, and role-specific interpretations of tactical behaviors. These developments move beyond traditional static graphs, offering richer and more nuanced representations of team interactions.

Despite this progress, several challenges remain. Research in this area continues to underrepresent key contexts, such as youth and women's football, and often overlooks defensive network structures. Future studies should prioritize longitudinal designs, single-team season analyses, multi-layer network models, and collaborative work with practitioners to enhance both theoretical depth and practical utility.

In sum, network analysis offers a scientifically grounded and operationally relevant approach to decoding the tactical and structural complexity of football. When appropriately contextualized and applied, it can support a range of performance domains, including coaching, match analysis, scouting, and strategic planning, ultimately contributing to the optimization of individual and team performance.

## Data Availability

The original contributions presented in the study are included in the article/Supplementary material, further inquiries can be directed to the corresponding author.

## Appendix

**SEARCH STRATEGIES ADOPTED SCOPUS**

(TITLE-ABS-KEY (football) OR TITLE-ABS-KEY (soccer) AND TITLE-ABS-KEY (network) OR TITLE-ABS-KEY (“social network analysis”) OR TITLE-ABS-KEY (“passing network”)) AND PUBYEAR > 2016 AND (LIMIT-TO (DOCTYPE, “ar”)) AND (LIMIT-TO (LANGUAGE, “English”))

**SPORTDiscus**

(football OR soccer) AND (network OR “passing network” OR “social network analysis)

Male, English, adults (19-44 years)

**PubMed (MEDLINE)**

((“football”[MeSH Terms] OR “football”[All Fields] OR “footballer”[All Fields] OR “footballer s”[All Fields] OR “footballers”[All Fields] OR “footballs”[All Fields] OR (“soccer”[MeSH Terms] OR “soccer”[All Fields] OR “soccers”[All Fields])) AND (“network”[All Fields] OR “network s”[All Fields] OR “networked”[All Fields] OR “networker”[All Fields] OR “networkers”[All Fields] OR “networking”[All Fields] OR “networks”[All Fields] OR “passing network”[All Fields] OR “social network analysis”[All Fields])) AND ((medline[Filter]) AND (male[Filter]) AND (alladult[Filter]) AND (2017:2024[pdat]))

**Web of Science**

(TS=(football) OR TS=(soccer)) AND (TS=(network) OR TS=(passing network) OR TS=(social network analysis)) AND Article(Document Types) AND English(Language)
