# Supplementary material for: Social network analysis in football: a systematic review of performance and tactical applications
Source: Front Psychol. 2025 Sep 17;16:1659603. doi: 10.3389/fpsyg.2025.1659603 (PMC12484057; doi:10.3389/fpsyg.2025.1659603)
Supplement: Supplementary file 1 [file Supplementary_file_1.docx]

Supplementary Material

# Supplementary Table 1. Analysis of the articles selected as a sample of the systematic review

| Study No | Author(s) | Journal Name | Sample | Aim | Network Variables | Key Findings |
| --- | --- | --- | --- | --- | --- | --- |
| 1 | Pina et al., 2017 | Frontiers in Psychology | 12 games of the Group Stage of UEFA Champions League 2015/2016 Group C | To test if the network metrics can be used to predict the outcome of offensive plays. | Network density, clustering coefficient, centralization | Low network density may be associated with a higher overall number of offensive plays but most of them are unsuccessful, and the high density was associated with fewer and/or longer offensive plays. High density may also be associated with fewer ball possession losses before the team reach the finishing zone. |
| 2 | McLean et al., 2017 | Theoretical Issues in Ergonomics Science | Analysis of the passing network prior to goals scored by teams from both EURO 2016 and COPA 2016 tournaments. | To test network analysis as a method to determine whether there were any differences between the teams from EURO and COPA. | Network density, cohesion, sociometric status, number of connections. | No significant differences between the EURO and COPA tournaments for all the measured variables. Although, the passing network of the EURO team were more connected, involved more players, occurred over a longer duration. |
| 3 | Ramos et al., 2017 | Frontiers in Psychology | 5 matches from a pool of 11 matches of the English Premier League season 2010-2011 | To apply the hypernetwork process to describe the behaviour of players and teams. | Hypernetworks | The results reveal that a micro-level the most frequently occurring simplices configuration is 1vs1; at a meso level, the simplices transformation near the goal depends on the variation of speed and direction in the players; at macro-level, simplices are connected from one another. |
| 4 | Peixoto et al., 2017 | Human Movement | 64 matches of the FIFA World Cup 2014 | To analyse the differences in the successful and unsuccessful interactions | Total links, network density, betweenness, indegree centrality and outdegree centrality | Lower levels in the network metrics can be associated with the offensive actions that result in goal. Longer possession chains may not be a success indicator. |
| 5 | Clemente & Martins, 2017a | International Journal of Computer Science in Sport | The best 16 teams that participated in the UEFA Champions League during the season 2015-2016 | To analyse the network properties of the best teams. | Total links, density, clustering coefficient | Teams that achieved the last phase of the competition had greater values of general network measures. Moderate correlations were found between possessions and network measures, suggesting that longer passing sequences involving more players in a more homogeneity manner. |
| 6 | Clemente & Martins, 2017b | Walailak Journal | The best 16 teams that participated in the UEFA Champions League during the season 2015-2016 | To analyse the centrality levels of the players and their variation between tactical system and playing position | Betweenness, indegree centrality and outdegree centrality | Central midfielders were considered the most prominent players in the offensive actions, presenting higher centrality values. Position of players is an important determinant of network of passes. |
| 7 | Arriaza-Ardiles et al., 2018 | Human Movement Science | 36 matches of a professional football team of the first Spanish division. | To apply a network theory model, to analyse the behaviour of a football team. | Clustering coefficient, closeness centrality and betweenness. | The total number of connections (passes and receptions) provides information to analyse the team. Metrics like, clustering and centrality, complement the information about the offensive play of the team. |
| 8 | Vivés et al., 2018 | Apunts Educación Físíca y Deportes | 32 matches from RCD Español, in the first division of the Spanish professional football league | To analyse the offensive behaviour of team RCD Español | Indegree and outdegree centrality. | The team attacked more when the ball was recovered on their own half, and the outside channels take greater importance in the attacking process of the team. |
| 9 | Yamamoto & Narizuka, 2018 | Physical Review | 6 matches from the Japanese Professional Football Leagues in 2016 | To investigate the growth of the passing network through the time, according to the Markov-chain. | Transition matrix | Teams with fewer number of passes tends to have greater error (transition probability). The team performance tends to be lower if the weighted passing network is highly centralized. |
| 10 | McHale & Relton, 2018 | European Journal of Operational Research | 380 matches of the English Premier League during the season 2012-2013. | To identify the key players in a football team. | Key players | It has been shown a model that can predict the probability of success of a pass in different areas of the pitch. The use of centrality measures can help to identify key players of a given team. |
| 11 | Clemente, 2018 | International Journal of Performance Analysis in Sport | 64 matches from FIFA World Cup 2018. | To test the relationship between the score outcome and network measures. | Density, total arcs, group clustering, reciprocity | Winners have lesser shots than losers and small increases in total arcs, network density group clustering and reciprocity. Meaningful moderate and positive correlations were found between goals scored and against and the total of arcs and network density during the matches that ended in a draw. |
| 12 | Mclean et al., 2018a | Human Movement Science | Goals scored at the 2016 European Football Championships | To determine the goal scoring passing network characteristics through the tournament, between the group stage and knock out stages, for the successful and unsuccessful teams. | Density, cohesion, sociometric status, within degree centrality, indegree and outdegree centrality. | There were no differences between the results of the group and knock out stage, between successful and unsuccessful teams, on the goal scoring passing network. There were scored more goals on fast, direct attacks with fewer passes. |
| 13 | McLean et al., 2018b | Human Movement | 21 matches from each two seasons of a professional football team playing in the Australian A league | To use network analysis to conclude the passing characteristics of playing position of a team | Outdegree centrality, indegree centrality, betweenness, closeness centrality. | Changes in the 1-4-2-2-2 to 1-4-2-3-1 formation had minimal impact in the general passing contributions of the team. Forwards presented more contribution to the team in the 1-4-2-2-2 formation compared with the 1-4-2-3-1. |
| 14 | Zhang et al., 2018 | International Journal of Geo-Information | 1 football match between Club Brugge KV and Standard Liege | To propose a hybrid approach to analyse the dynamic interactions in movement data | Attraction pattern, stability pattern, avoidance pattern, degree centrality, betweenness, closeness centrality, | Multi-Temporal Scale Spatio-Temporal Network and the association with centrality measures provides a multi-scale, network-based view of the interactions between players, allowing for both individual and collective analysis of movement patterns. |
| 15 | Oliveira & Clemente, 2018 | Journal of Physical Education and Sport | Analysis of 6 matches of a team playing on the UEFA Champions League during the season 2017-2018 | To analyse the relationship between distance covered and network properties | Distance covered, network density, total arcs, goals scored and suffered, total attempts, ball possession, passes  attempts and passes completed | Lower overall distances covered are strongly associated with a higher number of goals conceded. Additionally, a smaller number of pass attempts and lower ball possession are moderately linked to greater distances covered. Lastly, a higher number of total shot attempts is moderately associated with greater distances covered by team players. |
| 16 | Kawasaki et al., 2019 | International Journal of Performance Analysis in Sport | Analysis of 9 official matches of Fagiano Okayama of Japan Professional Football League Division 2 during the 2016 and 2017 seasons | To create a passing network based on the measurement of the pass position | Number of passes, total links, degree centrality, scaled connectivity and clustering coefficient | The nodes’ location was determined by clustering the positions of a passer and a receiver with respect to successful asses. The edges indicated the passes between different clusters. The network metrics analysed in this study indicate the relative level of the number of successful passes. |
| 17 | Mclean et al., 2019 | Science and Medicine in Football | 22 matches of the 2016-2017 competitive Australian A League season | To investigate the intra-team communication, and the relationship between the intra-team communication and passing of a professional football team, using a social network analysis approach | Density, edges, cohesion, sociometric status | The team was highly connected and cohesive for intra-team communication, but the teams passing was less connected and cohesive. |
| 18 | Mclean & Salmon, 2019 | Science and Medicine in Football | 2018 UEFA Champions League Final between Real Madrid and Liverpool | To investigate the broken passing links in football teams using network analysis and metrics | Network edges, density, percentage outdegree centrality (%  ODC) and percentage indegree centrality (%IDC) | Liverpool had a denser broken passing network and more broken network edges. The use of network metrics enables the researchers to identify the most prominent players involved in the broken passing links. |
| 19 | Diquigiovanni & Scarpa, 2019 | Statistical Modelling | 380 matches of the Italian Serie A TIM during the season 2015-2016 | To compare different playing styles of football teams | Nodes, edges | There were detected 15 playing styles. The teams that attacked through the lateral zones of the field had a good effect on the number of goals. The Dixon and Coles model doesn’t allow the prediction of the result of a game. The playing styles are only available at the end. |
| 20 | Praca et al., 2019 | Frontiers in Psychology | 14 matches of the knockout stage of the 2018 FIFA World Cup. | To analyse the influence of match status on team’s cooperation patterns and players prominence according to the positions | Density, clustering coefficient, degree centrality, page rank, degree prestige | Teams don’t change macrostructures according to match status. The microstructures showed changes in players behaviours according playing positions. The metrics, centrality and prestige, their levels in players of different positions indicated a more direct style in winning and a more build-up style in losing. |
| 21 | Korte et al., 2019 | Frontiers in Psychology | 70 professional football matches from 1 and 2 German Bundesliga during the season 2017-2018 | To identify dominant intermediary players, applying a play-by-play network. | Flow centrality, flow betweenness, weighted betweenness | The central defenders were identified as dominant and intermediary players in unsuccessful plays. And central midfielders are intermediary players in successful plays. |
| 22 | Castellano & Echeazarra, 2019 | Journal of Sports Science | 36 matches from 2 teams playing in the Spanish first division. | To analyse the centrality measures of the players and correlate with the physical demands when the team was in possession of the ball | Degree centrality,  betweenness centrality, degree prestige, closeness centrality and  page rank. | The best placed team showed higher values in the centrality metrics, total distance was higher in the best placed team, there were no statistical correlation between the level of centrality and physical demands, specifically in the best placed team. |
| 23 | Aquino et al., 2019 | International Journal of Performance Analysis in Sport | 61 matches from the 2018 FIFA World Cup | To investigate the possible variations of ball possession, match running performance, player prominence, team network according to match outcome and playing formation. | Indegree, outdegree, closeness centrality, betweenness, eigenvector, density and clustering coefficient | Most of the variables didn’t effect on the match outcome. The 1-4-2-3-1 tactical system was the most frequently used, and the teams who played in this system had greater values of ball possession. The relationship between player prominence and total distance covered in possession were position dependent. |
| 24 | Clemente et al., 2019 | Frontiers in Psychology | 64 matches of the 2018 FIFA World Cup | To analyse the variations of the network between close and unbalanced scores and, compare the centrality levels between playing positions | Degree prestige and degree centrality | The central defenders and defensive midfielders had greater levels of centrality in won close scores than in unbalanced. The defensive midfielders were the most recruited and who contributed more to the passing sequences. |
| 25 | Pereira et al., 2019a | Journal of Sports Engineering and Technology | Analyse the performance of Club Atlético de Madrid during the 2016-2017 season | To development and validation of the Golden Index formula | Betweenness centrality, influence rage closeness centrality, page rank prestige | The Golden Index formula, able to identify the individual contribution in the attacking phases of the team. The player Koke (midfielder) was identified as the Golden Player of the team. He presented the higher values in the network metrics, higher ratio in variables associated with the pass, and h was also the team player with more assists. |
| 26 | Buldú et al., 2019 | Scientific Reports | Analysis of Guardiola´s team, FC Barcelona, during the season 2009-2010. Analysis of 380 matches of “La Liga” | To analyse the differences in the network metrics and identify those parameters that change before or scoring or receiving a goal | Centroid, clustering coefficient, largest eigenvalue, algebraic connectivity, eigenvector, 50 pass network time | The network metrics were higher in FC Barcelona’s team compared to the rest of the teams. The number of passes benefits the network properties. |
| 27 | Pereira et al., 2019b | Motriz: Revista de Educação Física | Eight Atlético Madrid matches | To use the Golden Index to identify the Golden Players of Atlético Madrid team, and how individual performance is influenced by the quality of the opposition | Betweenness  centrality; influence range closeness centrality and  page rank | Golden Index formula allows to understand which players contribute the most for the offensive process of the team. The players Koke and Griezmann were identified as the Golden Players of the team, but the quality of the opponent may affect the results as well the attacking plays of the team. |
| 28 | Wiig et al., 2019 | International Journal of Computer Science in Sport | Four complete seasons of the Norwegian first division | To understand the players contributions and passing patterns by evaluating the pass difficulty, risk and potential | Closeness centrality, betweenness centrality, page rank, clustering coefficient | The use of network weights helps to identify the key passers and pass recipients. Defenders may rank highly in terms of pass difficulty as well in metrics like page rank. Attacking midfielders and strikers rank higher in pass potential, in the attacking plays that lead to goal attempts. |
| 29 | McLaren & Spink, 2020 | International Journal of Sport Communication | Two competitive adult male soccer teams. | To compare the network of member information exchange against a team performance outcome | Degree centrality and density | The winning team demonstrated an information exchange network with higher average individual degree centrality and network density. |
| 30 | Herrera-Diestra et al., 2020 | Chaos, Solitons and Fractals | FC Barcelona during the season 2009-2010 (“*La Liga*”) | To analyse the organization of the pitch networks of FC Barcelona (2009-2010) | Pitch networks, clustering coefficient, largest eigenvalue of the adjacency matrix and algebraic connectivity | The results indicated that the number of triangles of the pitch networks were higher in Barcelona team compared to rivals. Barcelona’s passing network was more robust than the rivals |
| 31 | Gama et al., 2020 | Nonlinear Dynamics, Psychology, and Life Sciences. | 10 matches between 10 professional team from the Portuguese League during the season 2010-2011. | To understand if there was a homogenous distribution in the number of passes made the member of a team, related to number of goals attempts and goals scored. | Entropy | It was found a existence of a pattern between an increase in the homogeneity of passing distributions and the attempts to scoring goals. Also, the homogeneous distribution of passes can predict with 45% accuracy when a goal attempt will occur. |
| 32 | Clemente et al., 2020 | Chaos, Solitons and Fractals | 64 matches from the 2018 FIFA World Cup | To analyse the variations of the network centrality between playing positions and, the influence of the teams scoring status on playing position | Degree centrality, degree prestige and reciprocity | The defensive midfielders had greater levels of degree prestige, being considered the most recruited by the teammates. The defensive midfielder and central defenders had the greater values of degree centrality contributing more for the ball possession of the team. |
| 33 | Sarmento et al., 2020 | Chaos, Solitons and Fractals | 16 matches from AS Monaco during the season 2016-2017. | To analyse the offensive process of AS Monaco through the combination of network methods and semi-structured interviews of 2 coaches from the technical staff. | Density, total arcs, clustering coefficient, arc reciprocity, dyad reciprocity, degree prestige, degree centrality, closeness centrality and proximity prestige | The defensive midfielder and the box-to-box, were the most prominent players in ball receptions and the central defender was the most recruited because of his ability to serve. |
| 34 | Martínez et al., 2020 | Entropy | 380 matches from Spanish national league (“*La Liga*”), during the 2017-2018 season | To obtain the spatial entropy of all passes of a team and how the players location on the field was related the entropy of his passes | Centroid, clustering coefficient, largest eigenvalue of the adjacency matrix, eigenvector centrality, 50 pass-network | Atlético Madrid and Valencia were the teams with the highest average spatial entropy. The spatial entropy changes according to the position of the players and, the passing network changes during the match and its evolution can be captured measuring the permutation entropy. |
| 35 | Yu et al., 2020 | International Journal of Sports Science & Coaching | 240 matches form the Chinese Super League during the 2017 season | To investigate the differences in matches performance between domestic and foreign player. | Neighbourhood connectivity, indegree, outdegree, stress centrality, partner, betweenness centrality and closeness centrality | Foreign players had an important role during the offensives plays of Chinese Super League teams. Foreign midfielders demonstrated higher values in passes out, outdegree and closeness centrality. |
| 36 | Martins et al., 2020 | Mathematics | 2015-2016 Champions League final | To present mathematical models for pattern analysis of a team, and to analyse the level of entropy in passing networks | Relative Transition Entropy and Network Transition Entropy | Real Madrid presented higher values for individual and team transition entropy, which indicated that higher values of unpredictability could bring teams closer to victory. |
| 37 | Aquino et al., 2020 | The Journal of Strength and Conditioning Research | 18 matches played in the 3rd Brazilian Division in 2017. | To analyse the independent and interactive effects of situational variables, opposition team formation, and playing position on running performance and network analysis in Brazilian professional soccer players during official match play. | Indegree, outdegree, betweenness, eigenvector, clustering, closeness, density and clustering coefficient | No interactive effects between team formation and playing position were observed for running and network variables; matches played at home or against weak opposition presented greater running demands and individual/global metrics of network analysis, match outcome demonstrated influence only for running performance with the team reporting higher values in matches won vs. lost; matches played at home or against weak opposition presented greater running demands and individual/global metrics of network analysis compared with their counterparts; match outcome demonstrated influence only for running performance with the team reporting higher values in matches won vs. lost; when the team competed in a 1‐4‐4‐2 formation, greater running demands were observed against a 1‐4‐4‐2 compared with a 1‐4‐2‐3‐1 formation; reduced values for running performance variables were reported in central defenders compared with peers in the other positions. Central/external midfielders reported greater closeness and betweenness centrality, outdegree, and eigenvector compared with central/ external defenders and forwards. |
| 38 | Y. Zhao & Zhang, 2020 | International Journal of Sports Science and Coaching | 1200 matches from the Chinese Super League during the 2014-18 seasons | To analyse the general network properties and centrality levels of the Chinese Super League during the 2014-18 seasons | Total Links, diameter, density, clustering coefficient, eigenvalue | Successful and home teams presented better results at links, diameter, density and clustering coefficient compared to unsuccessful teams or visiting teams. Winning teams presented lower density and clustering compared to the losing ones. Successful and winning teams presented a high level of passes and eigenvalues during the matches. |
| 39 | Medina et al., 2021 | Chaos, Solitons and Fractals | 380 matches from Spanish national league (“*La Liga*”), 2012-2013 season | To present an approach to determine the statistical role of the passing network in the performance of a team | Vertex degree, betweenness and entropy | Providing information about the time dependent game-average network provides to explain the outcome of a match. More finding reveal that the league champion had a larger connectivity. |
| 40 | Goncalves et al., 2021 | Human Movement | Analysis on 16 elite players during 14 matches in the 2018 1^st^ São Paulo State Championship | To understand the effects of match location,  quality of opposition, match outcome and playing position on load  parameters and players’ prominence, and to verify the relationships between load parameters  and players prominence | Indegree, outdegree. closeness centrality, betweenness and eigenvector | The players presented higher values in betweenness centrality and running outputs in matches against strong vs weak rivals. During the wins the players presented higher running demands and closeness centrality. Forwards presented reduced values of internal and external load and closeness centrality. Midfielders presented greater eigenvector values. |
| 41 | McLean et al., 2021 | Journal of Human Kinetics | 25 football players from the same club competing in the Australian A league | To use social network analysis to determine the playing positions that contributed more to beneficial intra-team communication compared to other team members; and to analyse the passing contributions of the individual playing positions | Indegree and outdegree | Central defender and midfielder were the ones that contributed the most to beneficial intra-team communication and passing, and the ones that achieved the higher player connectivity index. |
| 42 | Martins et al., 2021 | Entropy | 2020-2021 UEFA Champions League Final | To use new mathematical models and network analysis to analyse a match | Index rating of passing of a node, passing variability | Chelsea presents more passing variability than Manchester City, who presented more stable passing patterns. Higher variability is associated to a more unpredictable type of patterns. |
| 43 | Ichinose et al., 2021 | Chaos, Solitons and Fractals | Data from 45 matches of the J1 league (Japan) | To analyse the robustness of a passing network | Average degree, average distance, average clustering coefficient, robustness, change of diameter, algebraic connectivity and largest cluster change | It was showed that the passing network were robust against errors but vulnerable to attacks. Despite removing the key players, Kawasaki’s network was distinct from the other teams. |
| 44 | Ievoli et al., 2021a | Knowledge-Based Systems | 96 matches of 32 teams that played the 2016-2017 UEFA Champions League, during the group stage | To determine if network properties and performance indicators are crucial for the match outcome | Diameter, reciprocity, assortativity coefficient, cliques, clustering coefficient, degree centrality, betweenness, eigenvector, diversity, average neighbourhood, centralization, betweenness centralization, page rank, degree centralization, hub and authority | Network variables like diameter, betweenness centralization, can be related to the level of the offensive actions and finalizations of a team. |
| 45 | Ievoli et al., 2021b | AStA Advances in Statistical Analysis | Data from the 2016-2017 UEFA Champions League. | To use the network information to show how it can predict the match outcome. | Pass accuracy, intensity, diameter, reciprocity, median of average nearest neighbours and third quartile of hub | Bayesian hierarchical model is useful to find the determinants of match outcomes. Variables like passing speed (number of passes in the temporal unit) can improve the propensity of scoring goals. |
| 46 | Immler et al., 2021 | Frontiers in Sports and Active Living | 92 matches between 2017 to 2020 from UEFA Champions League | To use of passing networks to compare the styles of play of Guardiola, Klopp and Pochettino teams | Density, clustering coefficient, average shortest-path length, mean centrality and largest eigenvalue | Guardiola team have a proper signature in passing dynamics, with more players connected in the plays, compared with Klopp and Pochettino. |
| 47 | Assunção et al. (2022) | Applied Sciences | 1 UEFA Champions League match | To study the interactions between players in the critical game moments | Betweenness, closeness, degree centrality, degree prestige, assortativity coefficient, density, network heterogeneity and reciprocity | Critical game moments can serve as specific key events to understand team dynamic through macro and micro-level. |
| 48 | Armatas et al., 2022 | Journal of Physical Education and Sport | 48 knockout matches from the 2018 Men World Cup, Women 2019 and u20 Men 2019 | To analyse the differences between offensive transitions using network analysis in the Men, Women and Men u20 at the World Cup matches of 2018 and 2019 | Indegree and outdegree centrality and closeness centrality | Men achieve higher goal scoring attempts than Women and Men u20. Women won a higher proportion of set play compared to the other two groups. Women reported a higher proportion of possession lost. Age and gender influence in the transition success. |
| 49 | Alves et al., 2022 | Retos | 7 matches made from the Portuguese national team during the 2016 European Championship | To analyse the Portuguese offensive action that ended in shot during the 2016 European Championship | Density, degree centrality and degree prestige | More passes were made during the group phase compared with knockout phase. More interaction was made from the midfielder and the forward, assuming their role of importance inside of the team. |
| 50 | Zhou et al., 2023 | Applied Sciences | 38 matches of Everton during the 2017-2018 season in the Premier League | To develop a passing network model that provides a more qualitative analysis of the performance of a team | Indegree, outdegree, page rank, clustering coefficient, betweenness centrality | Using the 1-4-3-3 is related with winning networks and emphasizes forward progression, and the 1-4-2-3-1 is related with loosing networks and with limited connection between players. Page Rank results indicated that midfielders and attackers have importance in the team passing flow. The improved passing network and team coordination index can provide a clearer insight of players contribution. |
| 51 | Cao, 2023 | Journal of Sport Sciences | 2018 World Cup Final between Croatia and France | To analyse the state dynamics of team passing networks using time windows, graph distance measure, clustering and network modelling | Network intensity, betweenness centralization, average clustering coefficient | Croatia consistent passing patterns led to more stable periods, meanwhile transition between states led to key match events. The dynamic transitions between states highlight adaptability but also periods of inefficiency. France lower ball possession but higher passing intensity was associated with effective attacking patterns leading to their success. |
| 52 | Gong et al., 2023 | Chaos, Solitons & Fractals | 1200 matches during 5 consecutive seasons from the Chinese Super League | To analyse the consistency and identifiability of the Chinese soccer teams playing in the Chinese Super League | Pitch passing network | Well ranked teams presented higher identifiability results compared with the bottom ranked teams. This revealed their capacity to control the game. Top six teams revealed higher consistency with stabled passing patterns. |
| 53 | Pan et al., 2024 | Science and Medicine in Football | Analysis of 256 matches from 2010 to 2022 World Cups | To investigate the network evolution during FIFA’s World Cups from 2010 to 2022 | Average degree, average weighted degree, density, diameter, modularity, statistical inference, average clustering coefficient, average path length, indegree, outdegree, weighted indegree, weighted outdegree, closeness, betweenness, authority, clustering and eigenvector | A possession-based approach was shown during the years 2010 and 2014, a more direct approach occurred in the year 2018 and the return of the possession style in the year 2022. High ranking teams tend to adopt a possession-based approach compared with lower ranking teams. The importance of goalkeepers and defenders increased during the times, showing the evolution of tactical philosophies. |
| 54 | Nath & Chowdhury, 2024 | Social Network Analysis and Mining | Analysis of the knockout matches of the UEFA Champions League finalist Bayern Munich and Paris Saint-Germain during the 2019-2020 season, and the matches of Real Madrid and Liverpool during the 2021-2022 season | To combine social network analysis with position-specific football metrics to analyse the team and players performance | Eigenvector centrality, closeness centrality | Through player factor we can identify the key players in each match. Winning teams showed high median values in player factor, which indicate the contribution of top performing players. Weighted edge closeness centrality measures the proximity between players, revealing insights into coordination and network efficiency. |
| 55 | Novillo et al., 2024 | Chaos Solitons & Fractals | 380 matches from the Spanish National League (“La Liga”) during the 2018-2019 season | To propose a multilayer network framework to analyse football matches | Eigenvector centrality and pitch networks | Midfield zone presented higher eigenvector centrality, demonstrated the importance in controlling the game. High leakage values were presented in the corners, indicating frequent possession lost. Teams with high switching factors present high pressing tactics. |

**2** Table 2. Quality assessment of the articles included.

| **Author(s)** | **1** | **2** | **3** | **4** | **5** | **6** | **7** | **8** | **9** | **10** | **11** | **12** | **13** | **14** | **15** | **16** |
| --- | --- | --- | --- | --- | --- | --- | --- | --- | --- | --- | --- | --- | --- | --- | --- | --- |
| Pina et al., 2017 | ✔ | ✔ | ✔ | X | X | n.a. | ✔ | ✔ | ✔ | X | ✔ | ✔ | n.a. | ✔ | ✔ | ✔ |
| McLean et al., 2017 | ✔ | ✔ | ✔ | X | X | n.a. | ✔ | ✔ | ✔ | ✔ | ✔ | ✔ | n.a. | ✔ | ✔ | ✔ |
| Ramos et al., 2017 | ✔ | ✔ | ✔ | ✔ | ✔ | n.a. | ✔ | ✔ | ✔ | X | ✔ | ✔ | n.a. | ✔ | ✔ | ✔ |
| Peixoto et al., 2017 | ✔ | ✔ | ✔ | ✔ | 0 | n.a. | ✔ | ✔ | ✔ | ✔ | ✔ | ✔ | n.a. | ✔ | ✔ | ✔ |
| Clemente & Martins, 2017a | ✔ | ✔ | ✔ | X | ✔ | n.a. | ✔ | ✔ | X | ✔ | ✔ | ✔ | n.a. | ✔ | ✔ | ✔ |
| Clemente & Martins, 2017b | ✔ | ✔ | ✔ | ✔ | ✔ | n.a. | ✔ | ✔ | ✔ | ✔ | ✔ | ✔ | n.a. | ✔ | ✔ | ✔ |
| Arriaza-Ardiles et al., 2018 | ✔ | ✔ | ✔ | X | X | n.a. | ✔ | ✔ | ✔ | X | ✔ | ✔ | n.a. | ✔ | ✔ | X |
| Vivés et al., 2018 | ✔ | ✔ | ✔ | X | X | n.a. | ✔ | ✔ | ✔ | X | ✔ | ✔ | n.a. | ✔ | ✔ | X |
| Yamamoto & Narizuka, 2018) | ✔ | X | ✔ | ✔ | X | n.a. | ✔ | ✔ | ✔ | X | ✔ | X | n.a. | ✔ | X | X |
| McHale & Relton, 2018 | ✔ | ✔ | ✔ | X | X | n.a. | ✔ | ✔ | ✔ | ✔ | ✔ | ✔ | n.a. | ✔ | ✔ | X |
| Clemente, 2018 | ✔ | ✔ | ✔ | X | X | n.a. | ✔ | ✔ | ✔ | ✔ | ✔ | ✔ | n.a. | ✔ | ✔ | ✔ |
| Mclean et al., 2018 | ✔ | ✔ | ✔ | ✔ | X | n.a. | ✔ | ✔ | ✔ | ✔ | ✔ | ✔ | n.a. | ✔ | ✔ | ✔ |
| McLean et al., 2018 | ✔ | ✔ | ✔ | ✔ | X | n.a. | ✔ | ✔ | ✔ | ✔ | ✔ | ✔ | n.a. | ✔ | ✔ | ✔ |
| Zhang et al., 2018 | ✔ | ✔ | ✔ | ✔ | X | n.a. | X | X | ✔ | X | ✔ | ✔ | n.a. | ✔ | ✔ | ✔ |
| Oliveira & Clemente, 2018 | ✔ | ✔ | ✔ | ✔ | X | n.a. | X | ✔ | ✔ | X | ✔ | ✔ | n.a. | ✔ | ✔ | ✔ |
| Kawasaki et al., 2019 | ✔ | ✔ | ✔ | X | X | n.a. | ✔ | ✔ | ✔ | X | ✔ | ✔ | n.a. | ✔ | ✔ | ✔ |
| Mclean et al., 2019 | ✔ | ✔ | ✔ | X | X | n.a. | ✔ | ✔ | ✔ | ✔ | ✔ | ✔ | n.a. | ✔ | ✔ | ✔ |
| Mclean & Salmon, 2019 | ✔ | X | ✔ | X | X | n.a. | ✔ | ✔ | ✔ | X | ✔ | ✔ | n.a. | ✔ | ✔ | ✔ |
| Diquigiovanni & Scarpa, 2019 | ✔ | ✔ | ✔ | X | X | n.a. | ✔ | ✔ | ✔ | X | ✔ | ✔ | n.a. | ✔ | X | X |
| Praca et al., 2019 | ✔ | ✔ | ✔ | ✔ | ✔ | n.a. | ✔ | ✔ | ✔ | ✔ | ✔ | ✔ | n.a. | ✔ | ✔ | ✔ |
| Korte et al., 2019 | ✔ | ✔ | ✔ | X | X | n.a. | ✔ | ✔ | ✔ | ✔ | ✔ | ✔ | n.a. | ✔ | ✔ | ✔ |
| Castellano & Echeazarra, 2019 | ✔ | ✔ | ✔ | ✔ | X | n.a. | ✔ | ✔ | ✔ | X | ✔ | ✔ | n.a. | ✔ | ✔ | ✔ |
| Aquino et al., 2019 | ✔ | ✔ | ✔ | ✔ | X | n.a. | ✔ | ✔ | ✔ | ✔ | ✔ | ✔ | n.a. | ✔ | ✔ | ✔ |
| Clemente et al., 2019 | ✔ | ✔ | ✔ | X | X | n.a. | ✔ | ✔ | ✔ | ✔ | ✔ | ✔ | n.a. | ✔ | ✔ | ✔ |
| Pereira et al., 2019 | ✔ | ✔ | ✔ | X | X | n.a. | ✔ | ✔ | ✔ | X | ✔ | ✔ | n.a. | ✔ | ✔ | ✔ |
| Pereira et al., 2019a | ✔ | ✔ | ✔ | X | ✔ | n.a. | 0 | ✔ | ✔ | X | ✔ | ✔ | n.a. | ✔ | ✔ | ✔ |
| Buldú et al., 2019 | ✔ | ✔ | ✔ | ✔ | ✔ | n.a. | ✔ | ✔ | ✔ | ✔ | ✔ | ✔ | n.a. | ✔ | ✔ | ✔ |
| Wiig et al., 2019 | ✔ | ✔ | ✔ | ✔ | ✔ | n.a. | 0 | ✔ | ✔ | ✔ | ✔ | ✔ | n.a. | ✔ | ✔ | ✔ |
| McLaren & Spink, 2020 | ✔ | ✔ | ✔ | ✔ | ✔ | ✔ | ✔ | ✔ | ✔ | X | ✔ | ✔ | n.a. | ✔ | ✔ | X |
| Herrera-Diestra et al., 2020 | ✔ | ✔ | ✔ | ✔ | ✔ | n.a. | ✔ | ✔ | ✔ | X | ✔ | ✔ | n.a. | ✔ | ✔ | X |
| Gama et al., 2020 | ✔ | ✔ | ✔ | X | X | n.a. | ✔ | ✔ | ✔ | X | ✔ | ✔ | n.a. | ✔ | ✔ | X |
| Clemente et al., 2020 | ✔ | ✔ | ✔ | X | X | n.a. | ✔ | ✔ | ✔ | X | ✔ | ✔ | n.a. | ✔ | ✔ | ✔ |
| Sarmento et al., 2020 | ✔ | ✔ | ✔ | ✔ | ✔ | n.a. | ✔ | ✔ | ✔ | X | ✔ | ✔ | n.a. | ✔ | ✔ | ✔ |
| Martínez et al., 2020 | ✔ | ✔ | ✔ | X | ✔ | n.a. | ✔ | ✔ | ✔ | X | ✔ | ✔ | n.a. | ✔ | ✔ | ✔ |
| Yu et al., 2020 | ✔ | ✔ | ✔ | ✔ | X | n.a. | ✔ | ✔ | ✔ | X | ✔ | ✔ | n.a. | ✔ | ✔ | ✔ |
| Martins et al., 2020 | ✔ | ✔ | ✔ | ✔ | ✔ | n.a. | ✔ | ✔ | ✔ | X | ✔ | ✔ | n.a. | ✔ | ✔ | X |
| Aquino et al., 2020 | ✔ | ✔ | ✔ | X | X | n.a. | ✔ | ✔ | ✔ | ✔ | ✔ | ✔ | n.a. | ✔ | ✔ | ✔ |
| Y. Zhao & Zhang, 2020 | ✔ | ✔ | ✔ | ✔ | X | n.a. | X | ✔ | ✔ | ✔ | ✔ | ✔ | n.a. | ✔ | ✔ | ✔ |
| Goncalves et al., 2021 | ✔ | ✔ | ✔ | ✔ | X | ✔ | ✔ | ✔ | ✔ | ✔ | ✔ | ✔ | n.a. | ✔ | ✔ | ✔ |
| Medina et al., 2021 | ✔ | ✔ | ✔ | X | ✔ | n.a. | ✔ | ✔ | ✔ | X | ✔ | ✔ | n.a. | ✔ | ✔ | ✔ |
| McLean et al., 2021 | ✔ | ✔ | ✔ | ✔ | ✔ | n.a. | ✔ | ✔ | ✔ | ✔ | ✔ | ✔ | n.a. | ✔ | ✔ | ✔ |
| Ichinose et al., 2021 | ✔ | ✔ | ✔ | ✔ | X | n.a. | ✔ | ✔ | ✔ | X | ✔ | ✔ | n.a. | ✔ | ✔ | ✔ |
| Ievoli et al., 2021a | ✔ | ✔ | ✔ | X | X | n.a. | ✔ | ✔ | ✔ | X | ✔ | ✔ | n.a. | ✔ | ✔ | X |
| Ievoli et al., 2021b | ✔ | ✔ | ✔ | X | X | n.a. | ✔ | ✔ | ✔ | X | ✔ | ✔ | n.a. | ✔ | ✔ | ✔ |
| Martins et al., 2021 | ✔ | ✔ | ✔ | ✔ | ✔ | n.a. | ✔ | ✔ | ✔ | X | ✔ | ✔ | n.a. | ✔ | ✔ | X |
| Immler et al., 2021 | ✔ | ✔ | ✔ | X | ✔ | n.a. | ✔ | ✔ | ✔ | ✔ | ✔ | ✔ | n.a. | ✔ | ✔ | ✔ |
| Assunção et al. 2022 | ✔ | ✔ | ✔ | ✔ | X | n.a. | ✔ | X | ✔ | X | ✔ | ✔ | n.a. | ✔ | ✔ | ✔ |
| Alves et al., 2022 | ✔ | ✔ | ✔ | ✔ | ✔ | n.a. | X | ✔ | ✔ | ✔ | ✔ | ✔ | n.a. | ✔ | ✔ | ✔ |
| Armatas et al., 2022 | ✔ | ✔ | ✔ | ✔ | ✔ | n.a. | X | ✔ | ✔ | ✔ | ✔ | ✔ | n.a. | ✔ | ✔ | X |
| Gong et al., 2023 | ✔ | ✔ | ✔ | ✔ | ✔ | n.a. | X | ✔ | ✔ | X | ✔ | ✔ | n.a. | ✔ | ✔ | ✔ |
| Zhou et al., 2023 | ✔ | ✔ | ✔ | ✔ | ✔ | n.a. | X | ✔ | ✔ | X | ✔ | ✔ | n.a. | ✔ | ✔ | ✔ |
| Cao, 2023 | ✔ | ✔ | ✔ | ✔ | ✔ | n.a. | X | ✔ | ✔ | X | ✔ | ✔ | n.a. | ✔ | ✔ | X |
| Pan et al., 2024 | ✔ | ✔ | ✔ | ✔ | ✔ | n.a. | X | ✔ | ✔ | ✔ | ✔ | ✔ | n.a. | ✔ | ✔ | ✔ |
| Nath & Chowdhury, 2024 | ✔ | ✔ | ✔ | ✔ | ✔ | n.a. | X | ✔ | ✔ | ✔ | ✔ | ✔ | n.a. | ✔ | ✔ | X |
| Novillo et al., 2024 | ✔ | ✔ | ✔ | ✔ | ✔ | n.a. | X | ✔ | ✔ | X | ✔ | ✔ | n.a. | ✔ | ✔ | X |

Abbreviations: ✔- fulfils the criteria; X - does not fulfil the criteria; n.a. - the criteria does not apply
